# Differential regulation of reactive oxygen species in dimorphic chloroplasts of single cell C_4_ plant *Bienertia sinuspersici* during drought and salt stress

**DOI:** 10.3389/fpls.2023.1030413

**Published:** 2023-04-20

**Authors:** Baris Uzilday, Rengin Ozgur, Tolga Yalcinkaya, Mustafa Cemre Sonmez, Ismail Turkan

**Affiliations:** Department of Biology, Faculty of Science, Ege University, Bornova, Izmir, Türkiye

**Keywords:** antioxidant defense, *Bienertia sinuspersici*, drought stress, reactive oxygen species, salt stress, single cell C4 photosynthesis

## Abstract

Single cell C_4_ (SCC_4_) plants, discovered around two decades ago, are promising materials for efforts for genetic engineering of C_4_ photosynthesis into C_3_ crops. Unlike C_4_ plants with Kranz anatomy, they exhibit a fully functional C_4_ photosynthesis in just a single cell and do not require mesophyll and bundle sheath cell spatial separation. *Bienertia sinuspersici* is one such SCC_4_ plant, with NAD-malic enzyme (NAD-ME) subtype C_4_ photosynthesis. Its chlorenchyma cell consist of two compartments, peripheral compartment (PC), analogous to mesophyll cell, and central compartment (CC), analogous to bundle sheath cell. Since oxidative stress creates an important constraint for plants under salinity and drought, we comparatively examined the response of enzymatic antioxidant system, H_2_O_2_ and TBARS contents, peroxiredoxin Q, NADPH thioredoxin reductase C, and plastid terminal oxidase protein levels of PC chloroplasts (PCC) and CC chloroplasts (CCC). Except for protein levels, these parameters were also examined on the whole leaf level, as well as catalase and NADPH oxidase activities, water status and growth parameters, and levels of C_4_ photosynthesis related transcripts. Many C_4_ photosynthesis related transcript levels were elevated, especially under drought. Activities of dehydroascorbate reductase and especially peroxidase were elevated under drought in both compartments (CCC and PCC). Even though decreases of antioxidant enzyme activities were more prevalent in PCC, and the examined redox regulating protein levels, especially of peroxiredoxin Q, were elevated in CCC under both stresses, PCC was less damaged by either stress. These suggest PCC is more tolerant and has other means of preventing or alleviating oxidative damage.

## 1 Introduction

Climate change is one of the phenomena that will define the 21st century. Its numerous effects include more widespread and stronger drought and salinity (Okur and Örçen, 2020; IPCC, 2021), both of which have a negative impact on growth, development, and yield of crops (Anjum et al., 2011). Hence, an examination of their effects from different angles and underlying mechanisms is essential. Oxidative stress caused by drought and salinity is one such angle, as it is shared in both of these plant stresses. It is mainly caused by reactive oxygen species (ROS) formation, such as superoxide (O_2_.^–^), singlet oxygen (^1^O_2_), hydrogen peroxide (H_2_O_2_), and hydroxyl radical (^•^OH) (Gill and Tuteja, 2010). In plants, chloroplasts, mitochondria, peroxisomes, and apoplast are main sites of ROS production in the cell (Gill and Tuteja, 2010; Mittler, 2017; Foyer and Hanke, 2022). At lower levels ROS cause signalling and redox regulation, while at higher levels they cause oxidative damage to redox signalling or other components of the cells (Sies, 2019). For example, NADPH oxidases (NOXs) produce O_2_.^–^ which is often scavenged to H_2_O_2_ in apoplast, and these ROS originating from NOXs participate in signalling, conferring systemic resistance to abiotic and biotic stresses, and they interact with other signalling systems, such as, Ca^2+^, phosphorylation, and lipid signalling (Miller et al., 2009; Suzuki et al., 2011). Enzymatic and non-enzymatic antioxidants in the cells are vital for keeping ROS levels in check (Kim et al., 2021). Enzymatic antioxidants are important in the sense that they are highly specific in substrate selection (Haida and Hakiman, 2019). Superoxide dismutase (SOD) scavenges O_2_.^–^ to H_2_O_2_ while peroxidase (POX), catalase (CAT), and ascorbate peroxidase (APX) scavenge H_2_O_2_ to H_2_O (Choudhury et al., 2013). Moreover, monodehydroascorbate reductase (MDHAR), dehydroascorbate reductase (DHAR), and glutathione reductase (GR) participate in ascorbate-glutathione cycle, regenerating ascorbate (MDHAR and DHAR) and glutathione (GR) (Gill and Tuteja, 2010). Other oxidative stress tolerance mechanisms include numerous molecules, among which are peroxiredoxin Q (PRXQ), which is a 2-Cys peroxiredoxin that shows peroxidase activity under oxidative stress (Lee et al., 2018; Telman et al., 2019), NADPH-dependent thioredoxin reductase C (NTRC), which contains a thioredoxin domain and provides the needed reducing power to 2-Cys peroxiredoxins (Cejudo et al., 2012; Lee et al., 2018), and plastid terminal oxidase (PTOX), which is a quinol oxidase that oxidizes reduced O_2_ and plastoquinol (PQ) to H_2_O, relaxing PQ pool and preventing ROS formation that originates from it (Krieger-Liszkay and Feilke, 2016).

C_4_ plants are regarded as more photosynthetically efficient compared to C_3_ plants. This efficiency is strongly attributed to the decreased photorespiration of these plants, which increases substantially in C_3_ plants under stressful conditions (Sage et al., 2012; Cui, 2021; Sonmez et al., 2022). To achieve the suppression of oxygenation reaction of ribulose-1,5-bisphosphate carboxylase/oxygenase (RuBisCO), C_4_ plants use a carbon concentrating mechanism (CCM), which accumulates CO_2_ around RuBisCO (Sage et al., 2012). This way of photosynthesis is often said to confer tolerance to stressors such as drought or salinity, yet C_4_ plants are found to be geographically increasing or decreasing with precipitation (Paruelo and Lauenroth, 1996), and C_3_ and C_4_ subspecies comparison of *Alloteropsis semialata* demonstrated C_4_ subspecies to be more sensitive to drought (Ripley et al., 2007). On the other hand, C_4_ photosynthesis was found to evolve salinity tolerance (halophytism) more frequently and this was statistically associated with C_4_ photosynthesis (Bromham and Bennett, 2014). These findings paint a complex picture and necessitate a more thorough examination of the C_4_ photosynthesis.

**Figure 1:**
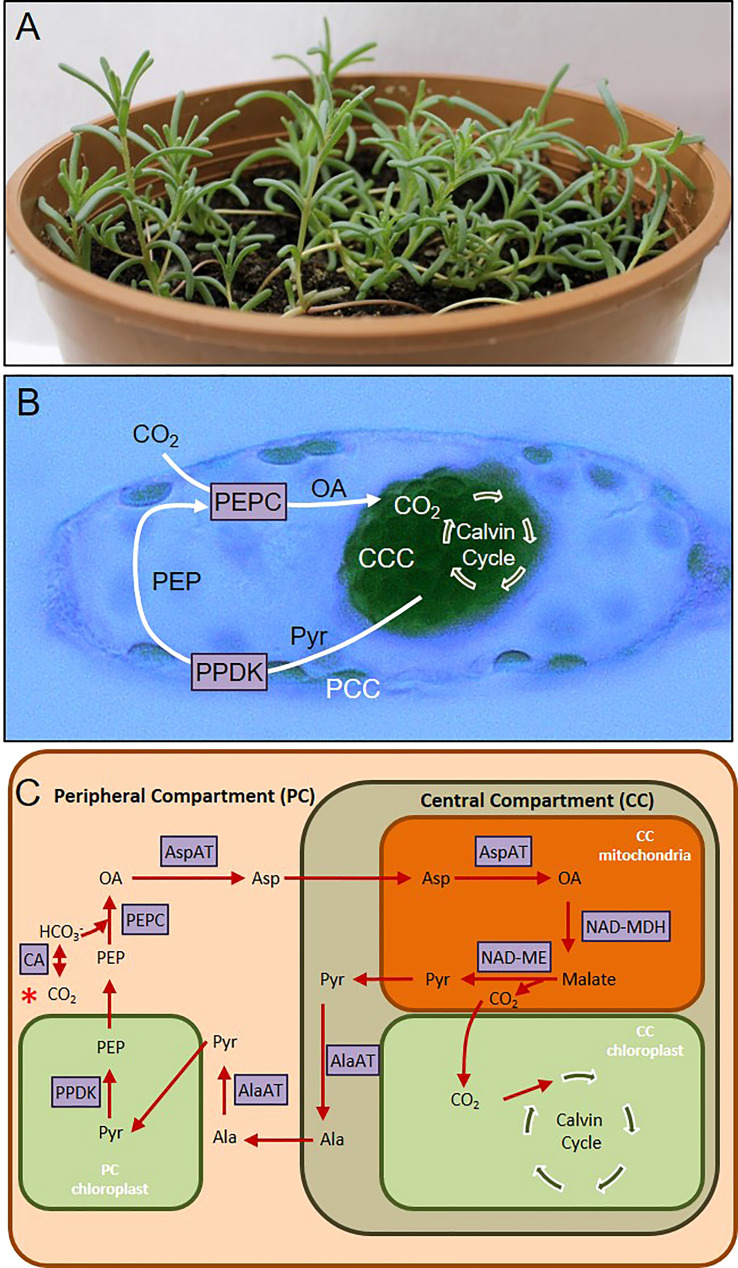
**(A)** *Bienertia sinuspersici* plant. **(B)** Cell micrograph showing central compartment (CCC) and peripheral compartment (PCC) chloroplasts. **(C)** Scheme summarizing single cell C4 (SCC_4_) pathway. Pathway starts with red asterisk on the left-hand side. AlaAT, Alanine aminotransferase; AspAT, Aspartate aminotransferase; CA, Carbonic anhydrase; NAD-MDH, NAD malate dehydrogenase; NAD-ME, NAD malic enzyme; OA, oxaloacetate; PEP, Phoshoenolpyruvate; PEPC, Phosphoenolpyruvate carboxylase; PPDK, Pyruvate phosphate dikinase; Pyr, Pyruvate.

For a long time, existence of C_4_ photosynthesis was associated with Kranz anatomy. In this organization, CO_2_ is initially turned into HCO_3_^-^ by carbonic anhydrase and then this molecule is fixed by phosphoenolpyruvate carboxylase (PEPC) in mesophyll cell to a four carbon organic acid, which is translocated to the bundle sheath cell. After translocation, a CO_2_ molecule is removed *via* decarboxylation, and finally RuBisCO in the bundle sheath uses this CO_2_ for Calvin cycle (Sharpe and Offermann, 2014). However, discovery of single cell C_4_ (SCC_4_) photosynthesis opened up new possibilities for examining C_4_ photosynthesis from different angles. In four species of Chenopodiaceae, namely *Suaeda aralocaspica, Bienertia cycloptera, Bienertia kavirense, Bienertia sinuspersici* (Sharpe and Offermann, 2014), the separation of PEPC and RuBisCO reactions are accomplished within a single cell, where the compartmentalization is achieved without plasma membrane or cell wall separation (Sharpe and Offermann, 2014). In *Bienertia sinuspersici* (**Figure 1A**), this is achieved by separating the peripheral compartment (PC) and central compartment (CC), with central vacuole surrounding the CC (**Figure 1B**). PEPC is found in PC, which is analogous to the mesophyll cell, and RuBisCO is found in CC, which is analogous to the bundle sheath cell. *B. sinuspersici* is a NAD-ME subtype SCC_4_ plant (Offermann et al., 2011; Offermann et al., 2015), and reactions of this metabolic pathway and enzymes involved are summarized in **Figure 1C**.

Despite C_4_ photosynthesis providing a possible pathway to increase crop yields, such as seen in the attempt of C_4_ rice project (Cui, 2021), there is still limited knowledge regarding the interactions of redox regulatory mechanisms and their response to stress in C_4_ plants (Turkan et al., 2018). While this is still a debate in classic Kranz C_4_ plants, and there are very few comparative studies that investigate redox regulatory mechanisms in C_3_ and C_4_ plants and their response to stress (Uzilday et al., 2014; Uzilday et al., 2012; Yildiz et al., 2021), there are no studies in single cell C_4_ plants like *B. sinuspersici*. This is an important gap in our knowledge, since elucidating the stress response of SCC_4_ plants has the potentialto lead to a better understanding of both Kranz type C_4_ and SCC_4_ plants, as we will be able to see the shared and differing responses of these plants. This is crucial for any attempt to incorporate any C_4_ trait to a C_3_ plant, as abiotic stressors such as drought and salinity are becoming more important in our world with a changing climate. For these reasons, we aimed to elucidate the antioxidant responses of *B. sinuspersici* whole leaves PC chloroplasts (PCC) and CC chloroplasts (CCC), comparatively under salt and drought stress.

## 2 Material and methods

### 2.1 Growth conditions and stress treatments

The seeds of *B. sinuspersici* were germinated at 25°C and germinated seeds were transferred to 1.5 L pots containing vermiculite:peat moss (1:1) mixture. Plants were grown at 25°C, 250 μmol m^-2^s^-1^ light intensity, 70% relative humidity in controlled growth chambers (16/8h light/dark regimes). Plants were watered every three days and 1 g L^-1^ Peters Professional fertilizer was added once a week (Offermann et al, 2011). *B. sinuspersici* is a halophyte and its growth is stimulated under mild saline conditions (Leisner et al., 2010), however, we choose to grow plants without salt supplementation to have stricter control on salinity stress experiment. Three months old plants were used for stress treatments. NaCl (400 mM) was treated to plants for salinity treatments (with 100 mM NaCl per day increments) and plants were treated for an additional week after reaching 400 mM NaCl. Drought stress was imposed as water withholding, which lasted for 10 d (see **Table 1** for procedure explaining stress treatment). Fully grown mature leaves were used for studies, since establishment of SCC_4_ cycle differs (Lara et al., 2008) among young, developing, and mature leaves.

**Table 1:** Method used for stress treatments.

Control	100 d of watering				
Salinity	90 d of watering	1 day 100 mM NaCl	1 day 200 mM NaCl	1 day 300 mM NaCl	7 days 400 mM NaCl
Drought	90 d of watering	10 d of water withholding			

### 2.2 Growth parameters, osmotic potential, and leaf relative water content

Shoot fresh weights and lengths were measured after treatments. To determine dry weights, samples were incubated at 72°C for 2 days and were weighted again. For root measurement, plants were removed from their pots with their substrate and vermiculite:peat moss mixture was washed away to reveal the root system.

Vapro Vapor pressure Osmometer 5520 was used to determine leaf osmotic potential. Six different plant leaves were used to assay osmotic potential. Leaf osmotic potential values were converted to MPa according to Santa-Cruz et al. (2002) by using coefficient of 2.408 × 10−3.

To measure leaf relative water content (RWC %), the fresh weights (FW) of leaves (n=6) were determined, then they were incubated in deionized water for 6h. After that, turgid weights (TW) were determined. Dry weights (DW) were measured after incubation in 80°C for 72h. The RWC percents were calculated using (FW-DW)/(TW-DW)x100 formula.

### 2.3 H_2_O_2_ and TBARS contents

Modified version of ferrous ammonium sulphate/xylenol orange (FOX) reagent was used to determine H_2_O_2_ according to Cheeseman (2006). Ethanol (1%) is added to FOX reagent to enhance its sensitivity to H_2_O_2_ (eFOX). Ice cold acetone including 25 mM H_2_SO_4_ was used for extraction, followed by 5 min 3000 g centrifuge at 4°C. A mix of 50/950μl sample/eFOX reagent was incubated for 30 min at room temperature, and absorbance at 550-800 nm were measured and calculated using standard curve. Thiobarbituric acid reactive substances (TBARS) content was determined according to Madhava Rao and Sresty (2000).

### 2.4 The activities of enzymes and isoenzymes

Samples (0.1 g) were grounded by liquid nitrogen and homogenized with 500 μl extraction buffer (50 mM Tris-HCl (pH 7.8), 0.1 mM ethylenediaminetetraacetic acid (EDTA), 0.1% (w/v) Triton-X100, 1 mM phenylmethylsulfonyl fluoride (PMSF), 1% polyvinyl polypyrrolidone (PVPP) (w/v)). Ascorbate (5 mM) was added to extraction buffer to measure the activity of APX. After centrifugation at 14000g for 10 min, supernatants were used for further analysis. Protein contents were determined by using bovine serum albumin (BSA) as standard, according to Bradford (1976). The activity of specific enzymes were given as U (mg protein)^-1^.

The activity of SOD (EC 1.15.11) was determined according to Beauchamp and Fridovich (1971) at 560 nm. CAT (EC 1.11.1.6) activity was measured as the initial rate of decomposition of H_2_O_2_ at 240 nm (Bergmeyer, 1970). The activity of POX (E.C1.11.1.7) was assayed, according to Herzog and Fahimi (1973), using 3,3-diaminobenzidine as chromophore and monitoring 3,3′-Diaminobenzidine (DAB) oxidation, which is accomplished by H_2_O_2_, at 465 nm. The activity of APX (EC 1.11.1.11) was determined as a decrease in absorbance at 290 nm (Nakano and Asada, 1981). The activity of GR (EC 1.6.4.2) was followed to determine oxidation of NADPH at 340 nm (Foyer and Halliwell, 1976). The activity of MDHAR (EC 1.6.5.4) was measured according to Arrigoni et al. (1981). The activity of DHAR (EC 1.8.5.1) was assayed according to Nakano and Asada (1981). The activity of NOX (EC 1.6.3.1) was determined at 470 nm according to Jiang and Zhang (2002).

Equal amounts of protein per sample were loaded to native polyacrylamide gel electrophoresis (Laemmli,1970). To separate isoenzymes of SOD, 4.5% stacking and 12.5% separating gels were used (Beauchamp and Fridovich, 1973). To differentiate the types of SOD isoenzymes, the gels were incubated with inhibitors of SOD before staining according to Vitória et al. (2001). To detect the isoenzymes of CAT, 7.5% separating gels were used (Woodbury et al., 1971) and the gels were subjected to 0.01% H_2_O_2_. Then they were washed with distilled water and incubated in 1% FeCl_3_ and 1% K_3_Fe(CN)_6_ staining solution. The electrophoretic separation of POX isoenzymes was performed with 10% separating gel, which were incubated in 200 mM Na-acetate buffer (pH 5·0) including 3 mM 3,3’-diaminobenzidine and 3% H_2_O_2_, for 30 min, according to Seevers et al. (1971). APX isoenzymes were assayed according to Mittler and Zilinskas (1993). The isoenzymes of GR were determined by incubating the 7.5% separating gels in a solution containing 10 mM TRIS-HCl (pH 7.9), 4 mM GSSG, 1.5 mM NADPH, and 2 mM 5,5′-dithiobis-(2-nitrobenzoic acid) (DTNB) for 20 min. The activity of GR was stained based on negative staining with 1.2 mM 3-(4,5-dimethylthiazol-2-yl)-2,5-diphenyltetrazolium bromide (MTT) and 1.6 mM phenazine methosulfate (PMS) for 10 min at room temperature (Hou et al., 2004). MDHAR isoenzymes were detected according to De Leonardis et al. (1995). NOX isoenzymes were separated with 7.5% native gels and stained with Tris-HCl buffer (50 mM pH 7.4) including 0.2 mM nitroblue tetrazolium (NBT), 0.1 mM MgCl_2_, 1 mM CaCl_2_, and incubated in dark for 20 min (Sagi and Fluhr, 2001). Gels were photographed with a gel imaging system and then analyzed with ImageJ software (Vilber Lourmat, Marne la Vallée, France).

### 2.5 Isolation of dimorphic chloroplasts

Isolation of dimorphic chloroplasts was carried out according to Offermann et al. (2011). For this, 5 g of leaves were homogenized with 15 ml protoplast buffer (5 mM MES-NaOH pH 5.8, 10 mM CaCl_2_, and Gly betaine (1.2 M for control and 1.8 M for salt and drought treated plants)). To remove the epidermis, the homogenate was passed through a 1 mm pore filter. Afterwards, it was centrifuged two times for 5 min at 4 g, and the pellet was re-dissolved in 6 ml of protoplast buffer. Cells were centrifuged at 9 g for 5 min and pellet was dissolved in 1 ml of cell wall digestion buffer (5 mM MES-NaOH, pH 5.8, 10 mM CaCl_2_, 2% Sumizyme C, 0.25% Macerse and Gly betaine). Cells were incubated for 1 h at 65 rotation per minute (rpm) on an orbital shaker. The protoplasts were centrifuged twice at 15 g for 5 min and the pellet was re-dissolved in 2 ml of protoplast resuspension buffer (20 mM Trisin-KOH, pH 8.4, 10mM EDTA, 10 mM NaHCO_3_ and Gly betaine). After centrifugation at 15 g, the protoplasts were treated with 500 µl of protoplast lysis buffer (20 mM Tricine-KOH pH 8.4, 10 mM EDTA, 10 mM NaHCO_3_, 1% BSA and Gly betaine). Protoplasts were disrupted by stepwise reduction of Gly betaine, hence buffer osmolality finally with buffer without Gly betaine. Afterwards, centrifugation was done at 40 g for 5 mins to separate the peripheral chloroplast and the central compartment. Peripheral chloroplasts in the supernatant were transferred to a clean tube. Lysis buffer of 6 ml was added to the pellet rich in the central compartment and passed through a 30 µm filter to remove intact protoplasts. The filtrate was centrifuged at 20 g for 5 mins and pellet was treated with cytoskeleton cleavage buffer (50 mM Tris-HCl pH 9.5, 5 mM MgCl_2_, 2.5 mM EDTA), and incubated on ice for 15 min. The structure of the microtubules was distorted due to the high pH and the central compartment chloroplasts were released. The obtained peripheral chloroplast fraction and central compartment chloroplast fraction were added to two step 40%-80% Percoll gradient. Lower cushion contained 2 ml of 80% Percoll, 5 mM MgCl_2_, and 2.5 mM EDTA in 20 mM HEPES-KOH pH 7.6. On the other hand, upper phase contained 5 ml of 40% Percoll, 1.2% PEG 6000, 0.4% Ficoll, 5 mM MgCl2, 2.5 mM EDTA in 20 mM HEPES-KOH pH 7.6. Each cushion contained Gly betaine compatible with respected treatment group (1.2 M for controls and 1.8 M for salinity and drought treatments). Finally, chloroplast preparations were laid upon the Percoll gradient and centrifuged at 2500 g for 6 min. After this step, dimorphic chloroplasts were obtained in pure form.

### 2.6 The isolation of RNA and qRT-PCR assay

RNA isolation was done with Qiagen RNeasy kit using 0.1 g leaf tissue. Total RNA was digested with DNase I to prevent genomic DNA contamination (Fermentas). Reverse transcription was done with 1 μg of DNAse I treated RNA using M-MuLV reverse transcriptase (New England Biolabs) to obtain cDNAs, which were used as templates for real-time quantitative reverse transcription polymerase chain reaction (qRT-PCR). The amount of RNA in each reaction was normalized to *EF1* gene of *B. sinuspersici*. qRT-PCR experiments were performed with Applied Biosystems StepOne Plus System using Power SYBR Green Master Mix (Applied Biosystems) with three technical replicates. The conditions for polymerase chain reaction (PCR) amplification were as follows: 95°C 5 min, 40 cycles at 94°C for 15 s, 60°C for 15 s, and 72°C for 30 s. The analyses of qRT-PCR data were done with StepOne Plus software. The reference point was control leaves and relative expressions in stress treated leaves were calculated with respect to control (set to 1). The list of primers is given in **Supplementary Material 1**.

### 2.7 Western blot analysis

Equal amounts of proteins were separated with sodium dodecyl sulfate polyacrylamide gel electrophoresis (SDS-PAGE), and separated proteins were transferred to polyvinylidene difluoride (PVDF) membranes using Bio-Rad TransBlot system. BSA (3%) was used to block the membranes which then were incubated with primary antibodies [Agrisera; PRXQ (AS05093), NTRC (AS07243), PTOX (AS163692)] overnight at 4°C. Membranes were then incubated with secondary antibody [Agrisera; donkey anti-rabbit IgG (AS101008)] conjugated with horseradish peroxidase for 1 h at room temperature. DAB (0.05%) and H_2_O_2_ (0.015%) in phosphate-buffered saline (PBS) (pH 7.2) solution was used for staining the bands of proteins. Vilber Lourmat gel imaging system was used to photograph the membranes and band intensities were calculated with ImageJ software. For quantification of bands in each gel see **Supplementary Material 2**.

### 2.8 Statistical analysis

The assays were done as two independent experiments, and each data point was the mean of three biological replicates (n = 6). The results were expressed as mean, and error bars were used for showing standard error of the mean ( ± S.E.M.). Groups were compared using Student’s t-test.

## 3 Results

### 3.1 Growth parameters and water relationships

**Figure 2:**
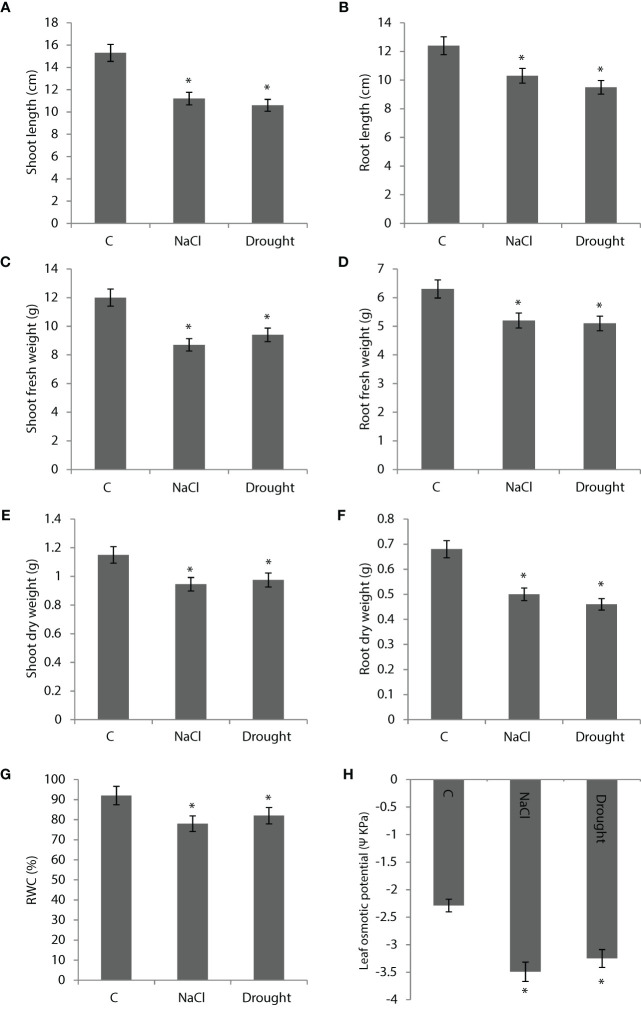
**(A, B)** Shoot and root length, **(C, D)** shoot and root fresh weight, **(E, F)** shoot and root dry weight, **(G)** relative water content (RWC) and **(H)** leaf osmotic potential of *Bienertia sinuspersici* plants treated with 400 mM NaCl or water withholding for 10 d. “*” indicates significant difference (p < 0.05) as compared to controls.

400 mM NaCl and drought applications decreased shoot length by 27% and 30%, respectively (**Figure 2A**). Root length was decreased by 17% and 24% with NaCl and drought applications, respectively (**Figure 2B**). NaCl and drought applications decreased shoot fresh weight by 28% and 22%, respectively (**Figure 2C**). In the same order, they decreased root fresh weight by 19% and 20% (**Figure 2D**). Shoot dry weight was decreased by 18% and 15% by NaCl and salinity, respectively (**Figure 2E**). NaCl and drought decreased root dry weight by 26% and 32%, respectively (**Figure 2F**). RWC was decreased by 14% and 11% by NaCl and drought, respectively (**Figure 2G**). Leaf osmotic potential was decreased by 53% and 42% by NaCl and drought, respectively (**Figure 2H**).

### 3.2 H_2_O_2_ and TBARS contents

**Figure 3:**
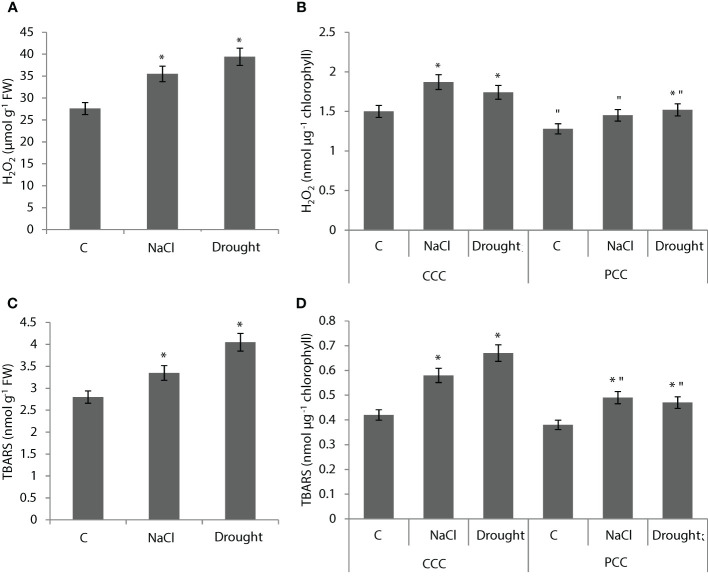
H2O2 **(A, B)** and lipid peroxidation **(C, D)** levels in whole leaves and dimorphic chloroplasts of Bienertia sinuspersici plants treated with 400 mM NaCl or water withholding for 10 d. * indicates significant difference (p < 0.05) as compared to controls. For central compartment chloroplasts (CCC) and peripheral compartment chloroplasts (PCC) groups are compared to their own controls. When effects of treatments are compared between CCC and PCC, significant differences are shown with “.

Whole leaf H_2_O_2_ content was increased by 29% and 43% with NaCl and drought applications, respectively (**Figure 3A**). H_2_O_2_ content was increased by 24% and 16% with NaCl and drought applications in CCC, respectively (**Figure 3B**). In PCC, H_2_O_2_ content was increased by 19% with drought application (**Figure 2B**). Whole leaf TBARS content was increased by 20% and 45% with NaCl and drought applications, respectively (**Figure 3C**). TBARS content of CCC was increased by 38% and 59% with NaCl and drought applications, respectively (**Figure 3D**). PCC TBARS content was increased by 28% and 24% with NaCl and drought applications, respectively (**Figure 3D**).

### 3.3 The activities of O_2_^–^related enzymes and isoenzymes in both whole leaf and dimorphic chloroplasts

**Figure 4:**
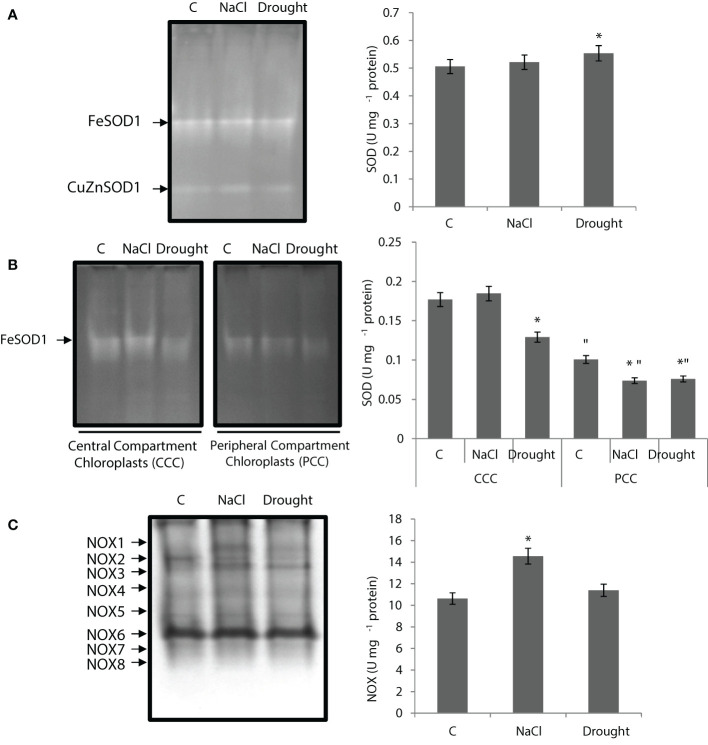
**(A)** Superoxide dismutase (SOD) native activity gel and total SOD activity of whole leaves. **(B)** SOD native activity gels and SOD activity of CCC and PCC. **(C)** NADPH oxidase (NOX) native activity gel and total NOX activity of whole leaves of *Bienertia sinuspersici* treated with 400 mM NaCl or water withholding for 10 d. “*” indicates significant difference (p < 0.05) as compared to controls. For CCC and PCC groups are compared to their own controls. When effects of treatments are compared between CCC and PCC, significant differences are shown with “.

SOD activity of the whole leaf didn’t change under salinity, while it increased by 10% under drought. Total of two isoenzymes of SOD were detected (FeSOD1 and CuZnSOD1), and FeSOD1 showed the highest activity and also increased with drought (**Figure 4A**). Total SOD activity of CCC didn’t show any statistically significant change under salinity, but it decreased by 30% with drought. SOD activity of PCC was decreased by 27% and 25% under salinity and drought, respectively. Only one FeSOD1 isozyme was detected in CCC and PCC (**Figure 4B**). The activity of FeSOD1 in CCC was decreased by drought stress.

NOX activity of the whole leaf increased by 37% under salinity, but it showed no statistically significant change under drought (**Figure 4C**). Eight isoenzymes (NOX1-8) were detected and NOX1 only appeared under stress. NOX6 was the isoenzyme that showed the highest activity. NOX2 activity decreased with stress applications (**Figure 1**).

### 3.4 The activities of H_2_O_2_ related enzymes and isoenzymes in both whole leaf and dimorphic chloroplasts

**Figure 5:**
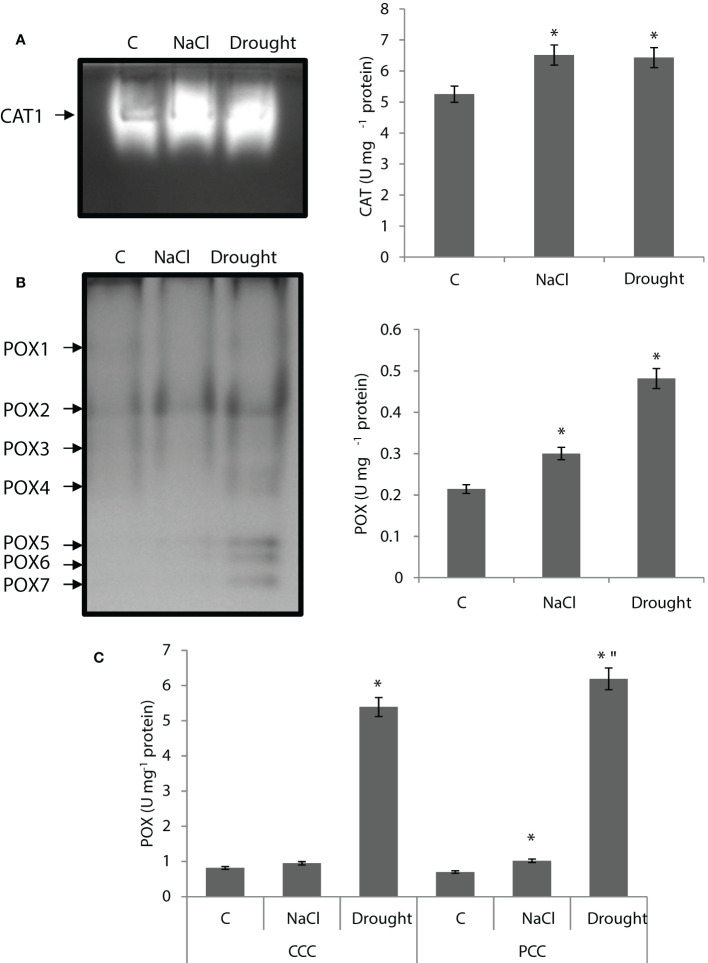
**(A)** Catalase (CAT) native activity gel and total CAT activity of whole leaves. **(B)** Peroxidase (POX) native activity gels and POX activity of whole leaves and **(C)** CCC and PCC of *Bienertia sinuspersici* treated with 400 mM NaCl or water withholding for 10 d. “*” indicates significant difference (p < 0.05) as compared to controls. For CCC and PCC groups are compared to their own controls. When effects of treatments are compared between CCC and PCC, significant differences are shown with “.

CAT activity of the whole leaf was increased by 24% and 22% by salinity and drought, respectively. Only one isozyme (CAT1) was detected and the activity of CAT1 isoenzyme was enhanced by both salinity and drought (**Figure 5A**).

POX activity of the whole leaf was increased by 43% and 125% by salinity and drought, respectively (**Figure 5B**). Seven isoenzymes (POX1-7) were detected in the assay while POX5, POX6, and POX7 only appeared with drought. POX2 was the isoenzyme with the highest activity (**Figure 5B**). Drought caused a severe increase in POX activity of CCC and PCC, for it was enhanced by 6.5 fold and 8.8 fold compared to controls, respectively, while salinity did not have this kind of effect on activity (**Figure 5C**).

### 3.5 The activities of water-water cycle enzymes and isoenzymes in both whole leaf and dimorphic chloroplasts

**Figure 6:**
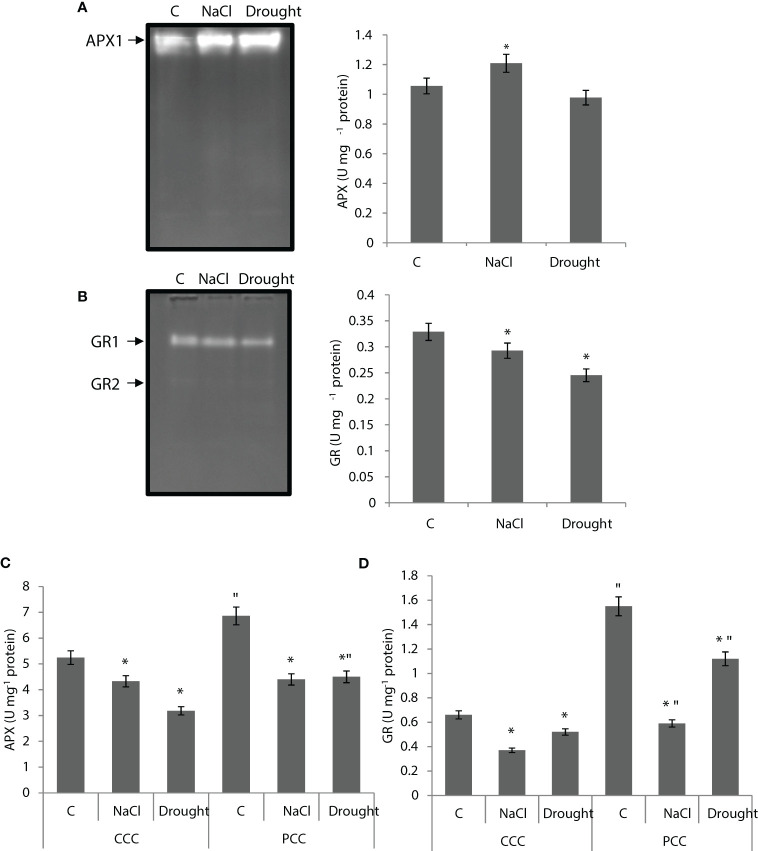
**(A)** Ascorbate peroxidase (APX) native activity gel and total APX activity of whole leaves. **(B)** Glutathione reductase (GR) native activity gel and GR activity of whole leaves. **(C, D)** APX and GR activities of CCC and PCC of *Bienertia sinuspersici* treated with 400 mM NaCl or water withholding for 10 d. “*” indicates significant difference (p < 0.05) as compared to controls. For CCC and PCC groups are compared to their own controls. When effects of treatments are compared between CCC and PCC, significant differences are shown with “.

APX activity of the whole leaf was increased by 14% under salinity, but it did not show any statistically significant change under drought (**Figure 6A**). One APX isoenzyme (APX1) was detected with native-page analysis. Salinity increased APX1 activity by 24%, while drought increased it by 31% (**Figure 6A**). GR activity of the whole leaf was decreased by 11% and 25% by salinity and drought, respectively. Two isoenzymes, GR1 and GR2, were detected and the activity of both isoenzymes was decreased under stress (**Figure 6B**). In the case of dimorphic chloroplasts, APX activity of CCC was decreased by 18% and 40% under salinity and drought, while APX activity of PCC was decreased by 38% and 35% with salinity and drought, respectively (**Figure 6C**). In CCC, the activity of GR was decreased by 44% and 22% under salinity and drought, respectively. It was decreased in PCC by 62% and 28% under salinity and drought, respectively. Only one isozyme was observed in both chloroplasts (**Figure 6D**).

**Figure 7:**
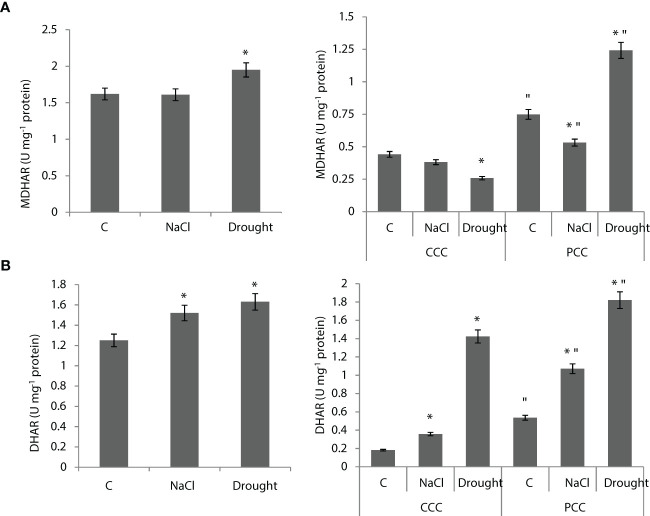
**(A)** MDHAR activity of whole leaves and CCC and PCC. **(B)** DHAR activity of whole leaves and CCC and PCC of *Bienertia sinuspersici* treated with 400 mM NaCl or water withholding for 10 d. “*” indicates significant difference (p < 0.05) as compared to controls. For CCC and PCC groups are compared to their own controls. When effects of treatments are compared between CCC and PCC, significant differences are shown with “.

MDHAR activity of the whole leaf didn’t show any statistically significant change under salinity, but it was enhanced by 20% under drought (**Figure 7A**). MDHAR activity of CCC didn’t show any change under salinity, but it decreased by 44% under drought. MDHAR activity of PCC was decreased by 29% under salinity, but it increased by 67% under drought. (**Figure 7A**). DHAR activity of the whole leaf was increased by 22% and 30% under salinity and drought, respectively. (**Figure 7B**). DHAR activity of CCC was increased by 94% and to 8-fold by salinity and drought, respectively. DHAR activity of PCC increased to 2-fold and 3.5-fold under salinity and drought, respectively (**Figure 7B**).

### 3.6 Expressions of C_4_ metabolism related genes

**Figure 8:**
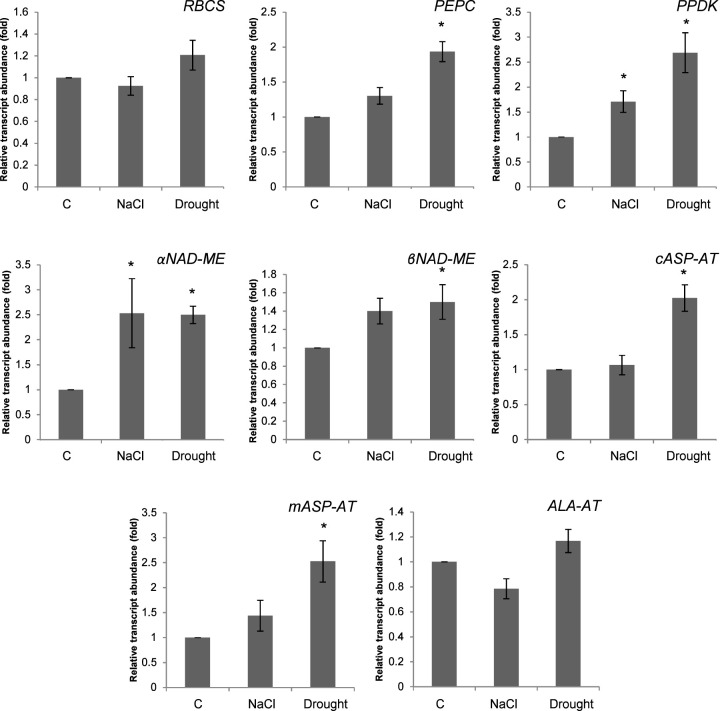
Expression of genes related to C_4_ cycle in *Bienertia sinuspersici* treated with 400 mM NaCl or water withholding for 10 d. “*” indicates significant difference (p < 0.05) as compared to controls.

The expressions of RuBisCO small subunit (RBCS), PEPC, PPDK, αNAD-ME, βNAD-ME, cASP-AT, mASP-AT, ALA-AT were determined under salinity and drought (**Figure 8**). Salinity enhanced the expressions of PPDK and αNAD-ME to 1.7- and 2.5-fold, respectively. Moreover the expressions of PEPC and PPDK increased to 1.9- and 2.7-fold as compared to controls, under drought. Drought also caused increases on the expressions of αNAD-ME and βNAD-ME. The expressions of *cASP-AT* and *mASP-AT* increased to 2- and 2.5-fold as compared to controls (**Figure 8**).

### 3.7 The levels of proteins related to alternative electron sinks in chloroplasts

**Figure 9:**
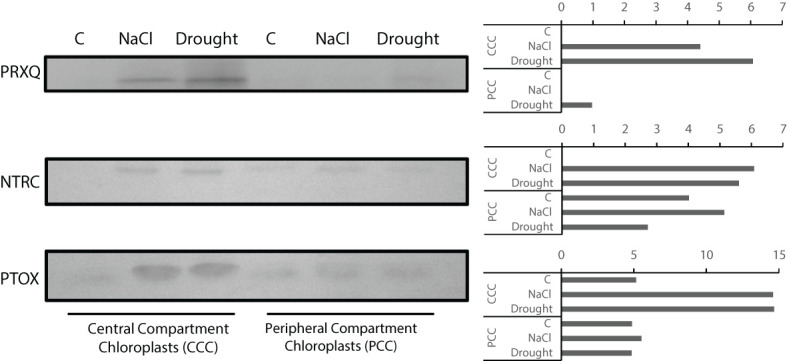
Levels of PRXQ, NTRC and PTOX proteins in CCC and PCC of *Bienertia sinuspersici* treated with 400 mM NaCl or water withholding for 10 d.

PRXQ, NTRC, and PTOX levels for CCC and PCC were examined. In CCC, PRXQ level increased under NaCl and drought, but it was more pronounced with drought. PRXQ was not detected in controls of CCC and PCC. In PCC, PRXQ was only detected with drought treatment (**Figure 9**). Levels of NTRC in both CCC and PCC were increased by NaCl or drought. Similarly, PTOX levels were increased by NaCl or drought both in CCC and PCC (**Figure 9**).

## 4 Discussion

In these experiments, shoot and root dry weights showed a decrease under both salinity and drought stress. Moreover, RWC and osmotic potential of leaves were also decreased, and fresh weight of shoots but not of roots was decreased. These all point to a loss of water in the plants, especially in the shoots, which leads to loss of turgor, and consequently, decreases in cell expansion and growth. The usual suspects of these stresses, decrease in water potential, and in the case of salinity, osmotic stress and ion toxicity, could be contributing factors (Anjum et al., 2011; Acosta-Motos et al., 2017). However, this situation could also partially be caused by increased leakiness of the CCC, the carbon concentrating compartment of *B. sinuspersici*, which is the chloroplast of CC that is analogous to the bundle sheath cell (BSC). BSC leakiness of C_4_ plants is known to increase under drought and salinity (Bowman et al., 1989), which may lead to overcycling of the C_4_ cycle, in which the C_4_ cycle fixes the same carbon that leaked out of BSC (Furbank et al., 1990). That may result in a decrease in the photosynthetic efficiency and a decrease in the growth of the C_4_ plant. This is supported by our gene expression analysis, which showed increased transcript levels of C_4_ cycle enzymes, as would be expected of plants which are overcycling under stress. Increases were especially prevalent and strong in drought treatment, which was appropriately harsher on the plant according to TBARS levels. However, increases in the transcripts of PEPC, PPDK, and NAD-ME could also be the result of non-photosynthetic functions of these enzymes, as PPDK, PEPC, and NADP-malic enzyme (NADP-ME) -which is similar to NAD-ME- are known to increase under abiotic stress even in C_3_ plants (Doubnerová and Ryšlavá, 2011).

In control plants, SOD activity was lower in PCC compared to CCC. This was also observed in the analogous compartment of PC, mesophyll cells (MCs), of NADP-ME C_4_ plants, when they were compared to BSCs (Doulis et al., 1997; Sundar et al., 2004). This suggests that SOD activity is required in carbon concentrating compartment of C_4_ and SCC_4_ plants. Control POX levels of two compartments weren’t substantially different in our experiment, but they were virtually non-existent in MCs yet substantially present in BSCs of sorghum, a NADP-ME C_4_ plant (Sundar et al., 2004). In contrast to our findings, Rosnow et al. (2014) found POX activity to be higher in CCC. This could be caused by differing growth conditions, as the carbon isotopic fractionation of *B. sinuspersici* changes depending on growing conditions, pointing to a shift in photosynthesis (King et al., 2011). This could affect the redox status which is also tied to the photosynthesis type of the plant (Turkan et al., 2018). Hence, this may be a probable explanation because growth conditions used by Rosnow et al. (2014) differ substantially from this study. Continuing on, APX, MDHAR, DHAR, and especially GR was higher in PCC. This effect was also observed for GR and DHAR in NADP-ME C_4_ plants (Doulis et al., 1997; Pastori et al., 2000 Sundar et al., 2004), in which these enzymes were higher in MCs. This suggests that GR and DHAR are important for the carbon assimilating compartment in C_4_ and SCC_4_ plants. Importantly, APX activity was higher in the BSCs of NADP-ME plants (Doulis et al., 1997; Sundar et al., 2004). This suggests a possible divergence of APX activity between NAD-ME and NADP-ME subtypes, since *B. sinuspersici* exhibits single cell NAD-ME subtype C_4_. But similarities mentioned previously should be interpreted with care, because the mentioned Kranz type C_4_ studies examined the antioxidant levels in the whole cell level, unlike our chloroplast focused approach. Moreover, these studies, including this one, were conducted on a very limited number of species, and while they provide some suggestions, they don’t necessarily provide a generalizable pattern for C_4_ subtypes or C_4_ itself. On the other hand, our study demonstrates that the antioxidant activity of organelles of C_4_ plants can differ from the whole leaf level. It should also be mentioned that Rosnow et al. (2014) found H_2_O_2_ level to be higher in PCC, while it was lower in PCC in our study. This could be caused by methodical differences, since the previous study used DAB staining, but the current study uses direct detection *via* xylenol orange. For example, DAB staining is known to be mediated by heme containing proteins, such as peroxidases (Daudi and O’Brien, 2012), and the variability of peroxidase activity in target compartments could have caused such an interference. Furthermore, it would make more sense for H_2_O_2_ content to be higher in CCC, because Offermann et al. (2015) found photosystem II (PSII) content of CCC to be higher than that of PCC. This can lead to H_2_O_2_ production *via* Mehler reaction through linear electron transport in the thylakoid membrane (Khorobrykh et al., 2015). On top of this, in NAD-ME subtype, granal index, the ratio of appressed thylakoid membrane to the total thylakoid membrane, is higher in bundle sheath chloroplasts, which are analogous to CCC. A *Bienertia* species different than the one used in this experiment was found to follow the same pattern for CCC, indicating higher linear electron transport in this compartment (Voznesenskaya et al., 2002; Voznesenskaya et al., 2003; Offermann et al., 2015).

Under NaCl treatment, the decrease or increase in antioxidant enzymes were always stronger in PCC. For SOD and MDHAR, CCC levels didn’t exhibit any statistically significant change while SOD and MDHAR activities decreased in PCC. For APX and GR, while both compartments showed a decrease, it was stronger in PCC. Therefore, when there was a decrease, it only manifested in PCC or was stronger in PCC. On the other hand, for POX, CCC didn’t exhibit any statistically significant change while PCC increased, and for DHAR while both compartment levels increased, it was stronger for PCC. Similar to the pattern observed for decreased activities, when there was an increase it only manifested in PCC or was stronger in PCC. This, PCC exhibiting stronger responses for both increases and decreases, leaves us with a complicated picture. Results for drought stress complicates it even more, since under drought, there was no discernible pattern for antioxidant enzymes.

The decreases in enzyme activity observed in chloroplasts for NaCl (SOD, APX, GR, MDHAR) and drought (SOD, APX, GR, MDHAR) can be partially explained by antioxidant system getting overpowered or damaged under stress. Ascorbate-glutathione pathway needs reducing power in the form of NAD(P)H to continue its function, and if ascorbate and glutathione aren’t reduced using NADPH, the pathway might collapse (Mittler et al., 2004; Choudhury et al., 2017). Since ascorbate, glutathione, and the four enzymes of the pathway (APX, GR, MDHAR, DHAR) are found in different compartments of the cell (Hasanuzzaman et al., 2019), this could at least partially explain the decreases of APX, GR, and MDHAR observed in CCC and PCC. The emphasis on compartment depletion is especially of importance, because some of these enzymes’ activities didn’t show a decrease on the whole cell level while they decreased in one or both of the dimorphic chloroplasts. Moreover, FeSOD and Cu/ZnSOD, which can be found in chloroplasts (Gill and Tuteja, 2010), are known to be inhibited by H_2_O_2_ (Sheng et al., 2014), and we observed increases in H_2_O_2_ in both chloroplasts for both stresses. But whether these increases in H_2_O_2_ are sufficient to explain the decreases in SOD activities is an important question.

The increase in DHAR under both conditions in both chloroplasts points to the importance of this enzyme for drought and salinity adaptation, but this increase could also simply be the result of ascorbate-glutathione pathway working as intended. This is because DHAR’s function is strongly attributed to its function in the ascorbate-glutathione pathway (Ding et al., 2020), and as explained before, activities of other enzymes of the pathway are affected negatively under stress. Thus while the adaptation response is to increase activity, other mechanisms could be decreasing the activities of other enzymes while DHAR is unaffected, perhaps because it uses GSH and not NADPH (Ding et al., 2020). The pronounced increase in POX activity under drought is also the result of stress adaptation. Furthermore, even though we may not have compared the two stresses in terms of magnitude, the increase of POX under drought is much more pronounced than under salinity. This can indicate that, POX is more important for combating drought than salinity in both chloroplasts of *B. sinuspersici*.

CCC exhibiting stronger increases in TBARS levels under both stresses shows that it’s more affected by both salinity and drought, as TBARS levels signify lipid peroxidation (Liu et al., 1997). Mechanisms other than enzymatic antioxidants must be at play, for there’s a discrepancy between enzyme activities and TBARS levels. A higher PSII level (Offermann et al., 2015) and higher granal index (Voznesenskaya et al., 2002; Voznesenskaya et al., 2003; Offermann et al., 2015) could be leading to higher ROS production in CCC (^1^O_2_ from PSII, and O_2_.^–^ from PSI due to Mehler reaction). Moreover, higher granal index would lead to increased amounts of thylakoid membranes in CCC. It has been previously demonstrated that thylakoid membranes have a high content of polyunsaturated fatty acids (PUFAs), which are prone to lipid peroxidation (Gill and Tuteja, 2010; Yalcinkaya et al., 2019). It was shown by Triantaphylides et al. (2008) that ^1^O_2_ is the major ROS responsible for lipid peroxidation, therefore higher ^1^O_2_ and PUFA at CCC might lead to higher lipid peroxidation. However, non-enzymatic antioxidants are also known to be involved in altering the redox status of the cell or a specific compartment (Gill and Tuteja, 2010; Krieger-Liszkay and Feilke, 2016), and their possible involvement can’t yet be ruled out.

When we take into account the H_2_O_2_ measurements, for salinity, considering that CCC showed a 24% increase in H_2_O_2_ level while PCC didn’t exhibit any statistically significant change, increase in TBARS level can be at least partially explained by the increase of H_2_O_2_ level in CCC. Yet for drought, H_2_O_2_ increases in both compartments are similar (16% vs. 19% for CCC and PCC, respectively), indicating other ROS are at play for the observed oxidative damage to lipids. Moreover, ultrastructural examination of NADP-ME (maize) and phosphoenolpyruvate carboxykinase (*Zoysia japonica*, *Amaranthus tricolor*) subtype C_4_ species showed mesophyll chloroplasts to be more vulnerable to salinity (Hasan et al., 2005; Omoto et al., 2010; Omoto et al, 2016). This might indicate that NAD-ME subtype C_4_ and/or SCC_4_ is different than other C_4_ subtypes, because the tolerance of these compartments seems to be reversed. Furthermore, under drought, lipid peroxidation of mesophyll cells was higher than that of bundle sheath cells in maize (Liu et al., 2022), indicating the same pattern is observed for drought too. However, paraquat treatment of maize, which produces O_2_.^–^ at photosystem I, showed that BSCs contained more protein carbonyl groups -which are formed as a result of oxidative stress- and were more sensitive than MCs (Kingston-Smith and Foyer, 2000). Thus, oxidative tolerance of C_4_ plants or at least of NADP-ME subtype might not be as straightforward as would be assumed from other studies. Our findings support this, showing that the tolerance of chloroplasts of the two compartments can be different from other studies.

The relative susceptibility of CCC is especially of importance for salinity, because antioxidant enzyme activities decreased more in PCC. All the more, CCC showed stronger increases for NTRC, PTOX, and especially PRXQ. PRXQ functions in redox regulation and it has peroxidase activity (Lee et al., 2018; Telman et al., 2019), NTRC feeds reducing power to 2-Cys PRX and alleviates oxidative damage (Cejudo et al., 2012), while PTOX can protect the plastoquinone pool from over-reduction, therefore inhibiting ROS generation (Krieger-Liszkay and Feilke, 2016). It is noteworthy to see these oxidative tolerance mechanisms amplified more in CCC under salinity, due to higher PSII levels and increased ROS production in this compartment. This implicates the existence of alternative mechanisms of preventing or alleviating oxidative damage in PCC under salinity. Among these candidates are, but not limited to, non-enzymatic antioxidants for they are also important for antioxidative tolerance (Gill and Tuteja, 2010; Hasanuzzaman et al., 2019).

## 5 Conclusions

In this work, we provide a snapshot of enzymatic antioxidants in whole leaves, CCC and PCC compartments, and their responses to drought and salinity in SSC_4_ plant *Bienertia sinuspersici*. Our data imply that, based on TBARS levels, PCC is more tolerant to drought or salinity induced oxidative stress than CCC. However, our data related to enzymatic antioxidants alone could not completely explain this tolerance and mechanisms other than enzymatic antioxidants might be at play. Increase in protein levels of PTOX, NTRC, and PRXQ might be caused by increased ROS production, leading to channeling of reducing power to protective mechanisms (NTRC-PRX) and ROS avoidance (PTOX) in CCC. Compatible with this, previous studies indicate higher ROS production in CCC, and ROS production dynamics (both ROS type and rate) in CCC and PCC, await further elucidation. Moreover, the possible role of non-enzymatic antioxidants needs further scrutiny and a subcellular metabolomics approach would help to further identify these molecules, to shed light onto tolerance mechanisms of this intriguing plant species.

## Data availability statement

The raw data supporting the conclusions of this article will be made available by the authors, without undue reservation.

## Author contributions

BU, RO, IT designed the experiments. BU, RO, TY performed the experiments. BU, RO, TY, MS analyzed the data. BU, TY, MS prepared figures. BU, RO, MS wrote the draft of the manuscript. BU, RO, IT reviewed and edited the final manuscript. All authors contributed to the article and approved the submitted version.
