# Supplementary material for: Differential regulation of reactive oxygen species in dimorphic chloroplasts of single cell C4 plant Bienertia sinuspersici during drought and salt stress
Source: Front Plant Sci. 2023 Apr 20;14:1030413. doi: 10.3389/fpls.2023.1030413 (PMC10157255; doi:10.3389/fpls.2023.1030413)
Supplement: Supplementary file 1 [file DataSheet_1.pdf]

# SOD native gel analysis of whole leaves

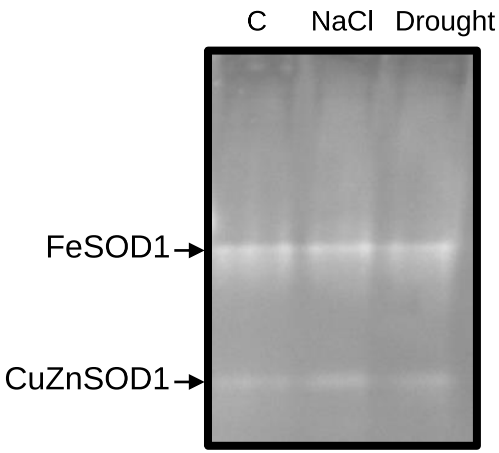


| | Control | NaCl | Drought |
|---|---|---|---|
| FeSOD1 | 100 | 108 | 116,4 |
| CuZNSOD1 | 100 | 98,3 | 110,4 |

Values are given as % of control

# Chloroplastic SOD native gel analysis of whole leaves

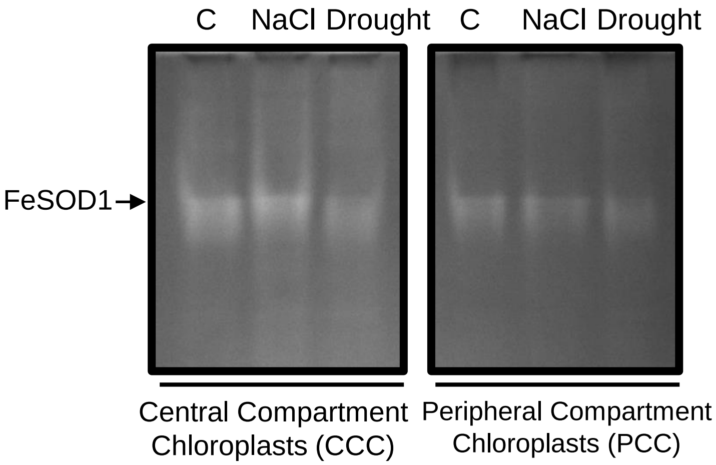


| | **Central Compartment Chloroplasts (CCC)** | | | **Periferal Compartment Chloroplasts (PCC)** | | |
|---|---|---|---|---|---|---|
| | Control | NaCl | Drought | Control | NaCl | Drought |
| FeSOD1 | 100 | 104,3 | 73 | 100 | 73,2 | 75,4 |

Values are given as % of control for each compartment (CCC or PCC)

# NOX native gel analysis of whole leaves

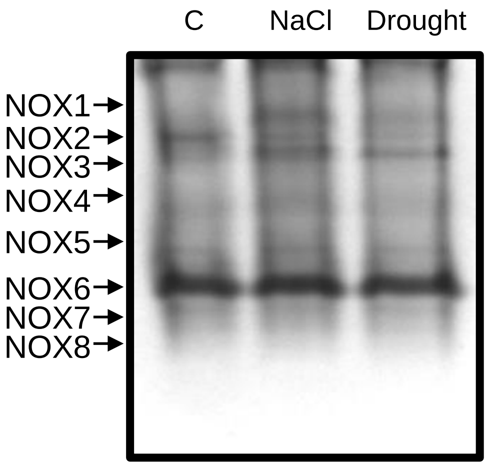


| | Control | NaCl | Drought |
|---|---|---|---|
| NOX1 | nd | 100 | 58,3 |
| NOX2 | 100 | 71,3 | 47,7 |
| NOX3 | 100 | 168,3 | 125,5 |
| NOX4 | 100 | 126 | 127,5 |
| NOX5 | 100 | 184,5 | 115,4 |
| NOX6 | 100 | 104,1 | 92,3 |
| NOX7 | 100 | 99,2 | 80,5 |
| NOX8 | 100 | 111,2 | 104,2 |

Values are given as % of control expect for NOX1 which can only be observed in salt and drought treated plants.

# CAT native gel analysis of whole leaves

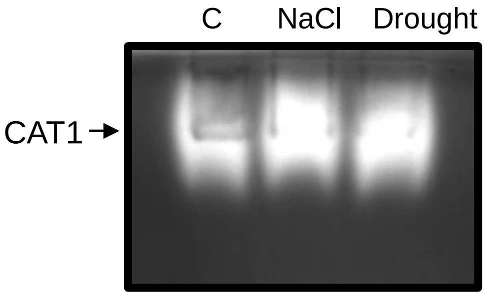


| | Cont | NaCl | Drought |
|---|---|---|---|
| CAT1 | 100 | 124 | 122,4 |

Values are given as % of control

# POX native gel analysis of whole leaves

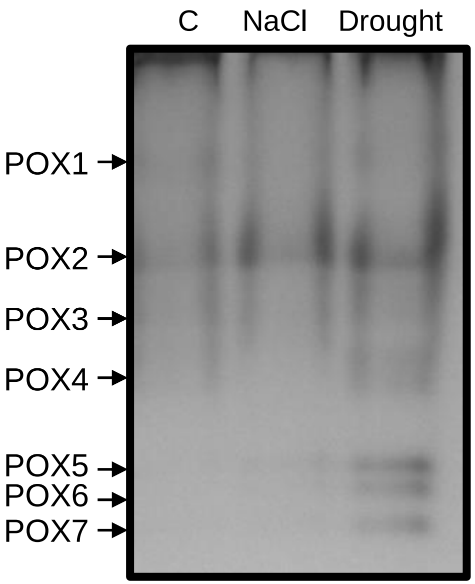


| | Cont | NaCl | Drought |
|---|---|---|---|
| POX1 | 100 | 113,4 | 143,6 |
| POX2 | 100 | 156,7 | 197,9 |
| POX3 | 100 | 135,4 | 166,9 |
| POX4 | 100 | 151,5 | 234,8 |
| POX5 | Nd | Nd | 100 |
| POX6 | Nd | Nd | 100 |
| POX7 | Nd | Nd | 100 |

Values are given as % of control expect for POX5-7 which can only be observed in drought treated plants

# APX native gel analysis of whole leaves

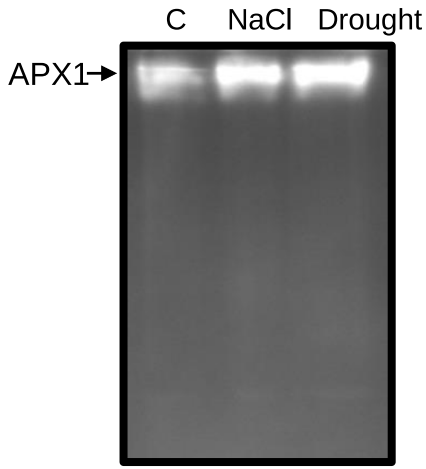


| | Cont | NaCl | Drought |
|---|---|---|---|
| APX1 | 100 | 124,2 | 131,3 |

Values are given as % of control

# GR native gel analysis of whole leaves

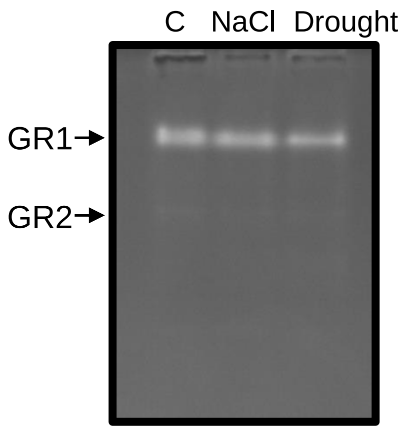


| | Cont | NaCl | Drought |
|---|---|---|---|
| GR1 | 100 | 95,8 | 78,21 |
| GR2 | 100 | 54,2 | 56 |

Values are given as % of control
